# Cardiovascular risk among Aboriginal and non-Aboriginal smoking male prisoners: inequalities compared to the wider community

**DOI:** 10.1186/1471-2458-11-783

**Published:** 2011-10-10

**Authors:** Robyn L Richmond, Kay A Wilhelm, Devon Indig, Tony G Butler, Vicki A Archer, Alex D Wodak

**Affiliations:** 1 School of Public Health and Community Medicine, University of New South Wales, Kensington, 2052. Australia; 2 Faculty of Medicine, University of New South Wales, Kensington, 2052, Australia; 3 Psychiatry, St Vincent's Hospital. Darlinghurst, 2010. Australia; 4 Faces in the Street, St Vincent's Health Urban Mental Health Research, St Vincent's Hospital. Darlinghurst, 2010. Australia; 5 Centre for Health Research in Criminal Justice, NSW Justice Health, Pagewood, 2035, Australia; 6 School of Public Health and Community Medicine, University of New South Wales, Kensington, 2052. Australia; 7 National Centre in HIV Epidemiology and Clinical Research, University of New South Wales. Kensington, 2052. Australia; 8 Alcohol and Drug Service, St. Vincent's Hospital, Darlinghurst, 2010. Australia

## Abstract

**Background:**

Cardiovascular risk factors (CVRF) were collected as part of a randomised controlled trial of a multi-component intervention to reduce smoking among male prisoners. Cross-sectional baseline data on CVRF were compared among smoking male prisoners and males of similar age in the general population.

**Methods:**

425 smoking prisoners were recruited (n = 407 in New South Wales; 18 in Queensland), including 15% of Aboriginal descent (mean age 33 years; median sentence length 3.6 years). We measured CVRF such as smoking, physical activity, blood pressure, risky alcohol use, symptoms of depression, and low socioeconomic status.

**Results:**

We found that 39% of prisoners had 3+ CVRF, compared to 10% in a general community sample of most disadvantaged men of a similar age. Significantly more Aboriginal prisoners had 3+ CVRF than non-Aboriginal prisoners (55% vs 36%, p < 0.01) and were twice as likely to have 4+ CVRF (27% vs 12%). In addition to all prisoners in this study being a current smoker (with 70% smoking 20+ cigarettes per day), the prevalence of other CVRF was very high: insufficient physical activity (23%); hypertension (4%), risky drinking (52%), symptoms of depression (14%) and low socioeconomic status (SES) (44%). Aboriginal prisoners had higher levels of risky alcohol use, symptoms of depression, and were more likely to be of low SES.

**Conclusion:**

Prisoners are at high risk for developing cardiovascular disease compared to even the most disadvantaged in their community and should be the focus of specific public health interventions.

**Trial Registration:**

This trial is registered with the Australian New Zealand Clinical Trials Registry ACTRN#12606000229572.

## Background

Cardiovascular disease (CVD) is a major problem in Australia with 3.4 million Australians affected [1] and 23% (800,000) of these also having an associated disability causing mild to-profound restrictions of activities such as self-care, mobility and communication. CVD carries the highest direct health-care expenditure of any disease group in Australia. This expenditure is expected to increase as the population ages. In Australia, CVD accounts for over three quarters of hospital stays among persons aged > 54 years or more and is responsible for around 1/3 of premature deaths. It is responsible for more deaths than any other disease, with costs exceeding 5 billion/year; i.e. 11% of the total healthcare expenditure in Australia since 2004 [2,3].

Risk factors for CVD include behavioural, biomedical, social, economic, psychological and cultural characteristics [2]. Estimating the prevalence of risk factors helps improve our understanding of the epidemiology of the disease and its prevention.

Some CVD risk factors have worsened over time. Physical inactivity increased over the past decade [3] while the average body weight increased by 6.5 kg for men and 7 kg for women in all age groups over the past 30 years [3,4]. Diabetes prevalence tripled since the late 1980s [3,5]. The cardiovascular risk factors of risky alcohol consumption, hypertension and high blood cholesterol have remained relatively static since 1993 [3]. National data on the latter two CVRF have not been updated since 2000 [3].

Death rates from CVD have declined by 76% from 831/100,000 population in 1968 to 202/100,000 in 2006 [6] due to improved public health interventions and medical treatment [7]. Public health strategies have improved cardiovascular health among Australians, resulting in lower mortality rates. For example, tobacco control strategies have lowered daily tobacco use in the general population to 17% [6].

However, this trend is not reflected in marginalised populations such as prisoners. In the 2009 New South Wales (NSW) Inmate Health Survey, 75% of male and 80% of female respondents were current smokers with 96% smoking hand rolled cigarettes (which have a higher nicotine and tar content than factory-made cigarettes) [8]. Tobacco has high value in prison and is used as a form of currency [9]. In 2002, the death rate from CVD in Australia was between 1.6 and 1.9 times higher among the most disadvantaged compared to the least disadvantaged areas [10].

This paper compares cardiovascular risk factors (CVRF) among male smoking prisoners with males of similar age and socio-economic background in the general population.

## Methods

Conduct of a randomised controlled trial of a multi-component smoking cessation intervention among prisoners in NSW and Queensland. This paper is a cross sectional survey which reports data from the baseline survey on CVRF of smoking male prisoners.

### Participants and recruitment

Male prisoners (n = 425) were recruited from 17 prisons across NSW (n = 407) and one prison in Queensland (n = 18). Female prisoners were not included as they constitute only 7% of the Australian prisoner population and their generally much shorter sentences [11] preclude participation in the intervention and follow up.

The study was promoted through posters, flyers, the prison health clinic staff and word of mouth. Study recruitment occurred between August 2006 and September 2009. Prisoners wishing to participate in a study of smoking cessation attended the prison health clinic, were assessed by a doctor and a research nurse administered the questionnaires. We assessed 1,751 male inmates who wished to stop smoking and excluded 1,315 as being ineligible. The main reasons for excluding these potential participants included: having a release date in the near future (35%), being on psychiatric medication or having a history of psychiatric illness (40%) and having a history of cardiac disease (5%). As the main study involved assessing the impact of an anti-depressant (nortriptyline) on a multi-component smoking cessation intervention, prisoners with a history of psychiatric illness or currently on psychiatric medication were also excluded. A further 11 participants were excluded who withdrew from the study before commencing the study medication, as they did not fulfill the intention-to-treat criteria.

### Questionnaire

The six CVRF measured in the larger clinical trial were current tobacco use, hypertension, insufficient physical activity, risky alcohol consumption prior to incarceration, current symptoms of anxiety and depression and low SES (defined as not completing year 10 of school).

Questions on smoking history, years of regular smoking, prior quit attempts and current smoking were collected. Blood pressure (BP) was measured at the baseline interview. Hypertension was defined as a systolic reading of 140 mmHg or more and/or diastolic reading of 90 mmHg or more. If an abnormal reading was obtained, blood pressure was repeated to check for accuracy. Physical activity was measured by self-report (number of times and number of minutes for a range of different exercise activities in the past four weeks) with 600 minutes per week (30 minutes/session, 5 times/week) as the benchmark. Risky alcohol consumption was defined as > 8 on Alcohol Disorders Identification Test (AUDIT) [12].

Anxiety and depression was assessed using the Kessler Psychological Distress Scale 10, a 10-item measure of psychological distress used to discriminate 'cases' of serious mental illness from 'non-cases', scores of ≥ 22 indicating anxiety or depression [13]. Although we screened out prisoners who had been diagnosed with current depression and were on antidepressant medication, we measured symptoms of depression as they constitute a significant independent risk factor for coronary heart disease [14].

Socioeconomic disadvantage is an independent CVRF [15]. This includes low income, low educational attainment, high levels of public sector housing, high unemployment and unskilled occupations [2,16]. Those with the lowest education level (not finishing year 10 at school) therefore did not have a qualification. The socioeconomic index divides the community into quintiles with the first quintile corresponding to the most disadvantaged and the fifth to the least disadvantaged [17].

Inmates who identified as Aboriginal or Torres Strait Islanders were considered Aboriginal.

### Informed consent and ethical approvals

Prisoners provided written informed consent to join the study. The study was independently approved by the UNSW Human Resources Ethics Committee, the NSW Department of Corrective Services Ethics Committee, Justice Health Ethics, Aboriginal Health and Medical Research Council ethics committee and Queensland Corrective Services Research Committee.

### Community comparison data

Cardiovascular risk factors for males aged 25-34 years in the general community were obtained directly from the Australian Bureau of Statistics (ABS) using a number of data sources. The majority of the information (smoking status, physical activity, alcohol use and depression) was obtained from the 2007/08 National Health Survey which included 20,788 participants [1]. Blood pressure for males aged 25-34 years in the general community was obtained from an analysis by the Australian Institute of Health and Welfare of the 1999-2000 Australian Diabetes, Obesity and Lifestyle (Ausdiab) study which included N = 11,247 participants [18]. Lastly, statistics about the proportion of men in the general community who did not complete year 10 was obtained from the 2006 Census of Population and Housing conducted by the ABS [18].

### Statistical Analysis

The clinical trial baseline questionnaires were double-key entered into an electronic database and the data was cleaned for potential errors. Statistical analysis was performed using SAS version 9.2 [19]. Chi-square descriptive statistics were used to compare differences between Aboriginal and non-Aboriginal study participants. ABS community comparison data was processed and cleaned using SAS 9.1 and then output through Supercross V4.3. Relative standard errors were derived using the jack-knife method with 60 replicate weights (ABS, personal communication).

## Results

### Demographics

The mean age of the 425 participants was 33 years, the median sentence length was 3.6 years and 64 (15%) participants were of Aboriginal descent.

### Individual cardiovascular risk factors

All participants were current smokers. In comparing the CVRF for Aboriginal and non-Aboriginal male prisoners with men of similar age in the general population (Table 1), prisoners were more than twice as likely to exercise sufficiently, were significantly more likely to have consumed alcohol at risky levels (especially Aboriginal inmates) in the 12 months pre incarceration, reported more depressive symptoms and poorer educational attainment.

**Table 1:** Cardiovascular risk factors among smoking male prisoners compared to males of similar age in the general population

Cardiovascular risk factors	Aboriginal % (n = 64)	Non- Aboriginal % (n = 361)	Total male prisoners in the study % (n = 425)	Males in the general population Aged 25-34 yr %
Current smoking	100	100	100	33^**a**^

Insufficient physical activity in last 4 weeks (≥ ul>30 mins 5 × per week)	23	23	23	68^**a**^

Risky alcohol use before entry to prison (score 8+ AUDIT)	69^**1**^	48	52	17^**a**^

Moderately high/high blood pressure (140+ systolic; 90+ diastolic)	2	5	4	7^**b**^

Current depression and anxiety symptoms (K10 score of 22+)	30^**1**^	12	14	11^**a**^

SES (education level ≤ Yr 9 or equivalent)	56^**2**^	41	44	5.6^**c**^

1. p < 0.01 when comparing Aboriginal to non-Aboriginal prisoners

2. p < 0.03 when comparing Aboriginal to non-Aboriginal prisoners

Sources:

a. ABS; National Health Survey Summary of Results 07-08. Cat #4364.0.

b. AIHW analysis of the 1999-2000 Australian Diabetes, Obesity and Lifestyle (AusDiab) study. http://www.aihw.gov.au/dataonline/riskfactors/index.cfm accessed 9/11/10

c. ABS 2006: 2006 Census of Population and Housing 2068.8

### Multiple cardiovascular risk factors

Table 2 compares the number of CVRF for prisoners with males of similar age in the most and least disadvantaged socioeconomic groups in the general population according to the ABS (2010) who customised their report for this paper, using the same criteria for disadvantage [18].

**Table 2:** Multiple cardiovascular risk factors among male prisoners (compared to males of similar age in the general population from most and least disadvantaged backgrounds)

Number of cardiovascular risk factors	Aboriginal male prisoners (n = 64) %	Non- Aboriginal male prisoners (361) %	Total Male prisoners (n = 425)%	% Australian males 25-34 yr* Most disadvantaged %	% Australian males 25-34 yr* Least disadvantaged %
0	0	0	0	27	30

1	9	22	20	41	46

2	36	42	41	22	21

3+	55	36	39	10	3

* Source: ABS, 2010 Customised Report [18].

Our study found that 166 (39%) prisoners had three or more CVRF, including 62 (14%) who had four or more CVRF. Of the Aboriginal prisoners, 55% had three or more CVRF and 27% had four or more which was significantly higher than rates for non-Aboriginal prisoners (36% and 12%, respectively). The CVRF profile of prisoners is considerably poorer than for men of a similar age from the most disadvantaged backgrounds in the general population (where 10% had three or more CVRF). One quarter of men in this age group, among the most and least disadvantaged in the general community, had no CVRF.

## Discussion

The study's key finding is that 39% of the male prison population had three or more CVRF compared to only 10% of disadvantaged men of similar age in the community. It is important to highlight that all prisoner participants in this study were smokers, so they all had at least one CVRF, while the community comparison included both smokers and non-smokers. Though this comparison is imperfect, it is not considered to be inappropriate as the proportion of male prisoners who are current smokers is 75%, compared to 16% in the general community, which suggests that most male prisoners do have smoking as a CVRF [8,20]. Further, we propose that these findings are likely to be an underestimate of the number of CVRF among prisoners as the study explicitly screened out prisoners with diagnosed psychiatric illness and/or cardiac disease. The 2009 NSW Inmate Health Survey recruited 996 male prisoners, of whom 19% reported that they had been told by a doctor that they had a heart problem [8]. This suggests that the CVRF profile of prisoners is significantly poorer than the findings reported here.

Cardiovascular risk factors for male prisoners in this study were similar to that reported in the 2009 NSW Inmate Health Survey (IHS). Both studies identified 23% of male prisoners with insufficient physical activity, similar rates for not completing year 10 (56% vs 52% IHS), similar rates for depression (30% vs 33% IHS) and for risky alcohol consumption (69% vs 63% IHS). As this study only recruited males who smoked tobacco, the proportion for that cardiovascular risk factor was 100%, compared to 75% of males in the IHS who reported they currently smoked tobacco. As this study also excluded men with a history of cardiac disease, the proportion of participants found with high blood pressure (2%) was significantly lower than found in the IHS (15%), which suggests that this study underestimates the extent of cardiovascular risk among male prisoners [20].

This study found that significantly more Aboriginal prisoners had three or more CVRF than non-Aboriginal prisoners. The burden of disease among Aboriginal Australians has been estimated to be 2.5 times greater than among non-Aboriginal Australians [21]. One reason for this is that Aboriginal people have a greater likelihood to have multiple risk factors such as tobacco smoking, physical inactivity, poor nutrition and risky alcohol consumption. These factors have been found to be higher among Aboriginal prisoners in NSW compared to non-Aboriginal prisoners [20]. Prisons are an important setting in which health interventions can be developed for hard to reach populations, such as Aboriginal people, who may not access health care in the community [22]. Improving the health of Aboriginal people in prison can contribute towards attempts by the Australian government to reduce gaps in health and well-being between Aboriginal and non-Aboriginal Australians [23].

This study also found that prisoners are at even higher risk for developing cardiovascular disease compared to males of similar age in the general population from the most disadvantaged quintile in our community. This suggests that the highly disadvantaged backgrounds of prisoners are more severe than is found among the most disadvantaged in the community. There is a direct relationship between the number of CVRF and the development of atherosclerosis, which correlates with reduced life expectancy and greater health care costs [17]. The cumulative effects of multiple risk factors may be additive or synergistic. A focus on moderate reductions in several risk factors is likely to have more benefit than focussing on achieving a major reduction in one factor [24].

### Limitations of our study

As our study was not primarily designed to investigate CVRF we did not measure all possible risk factors. We did not collect objective data on diabetes or cholesterol or weight (to determine presence of overweight/obesity, a major CVD risk factor). Another limitation is that a second blood pressure reading was only taken if an abnormal first blood pressure reading was obtained. Therefore our ascertainment of CVRF is subject to the vagaries of self-report. The 2009 NSW Inmate Health Survey reported that 55% of male prisoners were found to be overweight or obese [8] so we would expect a similar proportion in our study to have been overweight or obese. Thus we have underestimated the prevalence of CVRF by not including overweight prisoners. Further, as this was part of a randomised control trial, our eligibility criteria excluded prisoners with current cardiac disease and those who were on current medication for a mental illness, including depression. Hence, a further limitation of our study is that prisoners with these cardiovascular risk factors were excluded from our study. Therefore, the number of risk factors among prisoners is an underestimation.

A further limitation is that the study participants were all smokers compared to only a portion of the general community sample of men. This may bias the results among prisoners to a higher risk group compared to the general community. However, as 75% of male prisoners are current smokers, this limitation is relatively consistent with the risk profile of prisoners [8].

The Framingham Risk Equation (FRE) is widely used to estimate cardiovascular risk. However, there are limitations with the FRE as it does not include some significant risk factors including overweight/obesity, physical inactivity, family history of CVD, socioeconomic status, psychological factors [25,26]. Further, the FRE has been shown to overestimate CV risk in populations with low CV mortality, but greatly underestimate risk among those with high mortality, including those who are socioeconomically deprived groups [27], such as prisoners and Indigenous people [15,28].

### Clinical and research implications

The CVD mortality rate among the most disadvantaged males in Australia was 112% higher compared to the least disadvantaged males [10]. Higher death rates from CVD are due to socioeconomic inequality between the most and least disadvantaged which has widened over the past decades [10,29,30]

Community studies have reported that CVD is 1.3 times more prevalent among Aboriginal Australians compared to non-Aborigines [31]. In our study we found that CVRF were more prevalent among prisoners, particularly Aboriginal prisoners, when compared to the most disadvantaged males in the general community of similar age. Excess mortality from CVD is likely to be even greater among prisoners.

Re-entry into the community can be a stressful experience involving adjustment to relationships and family, seeking employment and housing. Several studies have assessed ex-prisoners' post-release death rates and have shown that they are at increased risk of death [32-34]. Mortality in Australian ex-prisoners released from custody was significantly higher compared to the NSW population [35]. Deaths were particularly high from mental and behavioural disorders, suicide, drug overdose and homicide. Ex-prisoners have been found to be 3.5 times more likely to die just after release compared to the general public from CVD, drug overdose, homicide and suicide [36]. The first 6-12 months following release from prison is a high-risk time. Released Aboriginal men in Western Australia (WA) have an almost 10 times greater risk of death than the general WA population and an almost three times greater risk of death compared with their Aboriginal peers residing in the community [32]. Suicide, high risk drug and alcohol consumption and motor vehicles accidents are the main causes of death.

## Conclusions

Although public health campaigns and strategies have had a major impact on reducing individual risk factors in the Australian community, they appear to have had very little impact on the high risk groups in prison. We found that male prisoners are at significantly higher risk from cardiovascular disease compared to the most disadvantaged of the same age in the general male population. Aboriginal prisoners are at even higher risk as they have significantly higher prevalence of risk factors. The burden of cardiovascular risk identified in our study is considerably higher than the general male population and is likely to be considerably higher than we estimated as we eliminated prisoners from our study who had certain risk factors for CVD, such as having a history of cardiac disease or psychiatric illness.

Correctional facilities should be a focus of public health interventions to improve cardiovascular health. Incarceration is an opportunity to improve physical activity. Inmates have high rates of exercise as shown in the comparison of physical activity with the general population of the same age. The high rates of CVRF among the prisoner population suggests the need to offer evidence-based behaviour change interventions so that inmates can begin their lives on release with safer levels of lifestyle factors. Prisoner specific public health interventions to improve cardiac health should be mindful of the sub populations that are over-represented in prison: those with low socioeconomic status and disadvantage, people with a mental illness, illicit drug users and Aboriginal Australians.

## Competing interests

The authors declare that they have no competing interests.

## Authors' contributions

RR, KW, TB and AW were involved in all aspects of this study from conceptualisation, funding, and development of questionnaire for baseline and follow-up assessments. DI and VA joined the study and made substantial contributions to data acquisition and statistical analyses. All authors have been involved in data interpretation, drafting the manuscript and revising it critically for important intellectual content; and have given final approval of the version to be published.

## Pre-publication history

The pre-publication history for this paper can be accessed here:

http://www.biomedcentral.com/1471-2458/11/783/prepub
